# Use of Artificial Intelligence Methods for Improved Diagnosis of Urinary Tract Infections and Urinary Stone Disease

**DOI:** 10.3390/jcm14144942

**Published:** 2025-07-12

**Authors:** Theodor Florin Pantilimonescu, Costin Damian, Viorel Dragos Radu, Maximilian Hogea, Oana Andreea Costachescu, Pavel Onofrei, Bogdan Toma, Denisa Zelinschi, Iulia Cristina Roca, Ramona Gabriela Ursu, Luminita Smaranda Iancu, Ionela Lacramioara Serban

**Affiliations:** 1 Department of Physiology, “Grigore T. Popa” University of Medicine and Pharmacy, 700115 Iasi, Romania; 2 Department of Preventive Medicine and Interdisciplinarity (IX)—Microbiology, “Grigore T. Popa” University of Medicine and Pharmacy, 700115 Iasi, Romania; 3 Department of Urology, “Grigore T. Popa” University of Medicine and Pharmacy, 700115 Iasi, Romania; 4 Department of Urology, Regional Institute of Oncology, 700483 Iasi, Romania; 5 Department of Morphofunctional Science, II, “Grigore T. Popa” University of Medicine and Pharmacy, 700115 Iasi, Romania; 6 Department of Obstetrics and Gynaecology, “Grigore T. Popa” University of Medicine and Pharmacy, 700115 Iasi, Romania; 7 Department of Surgery II, Emergency Medicine, “Grigore T. Popa” University of Medicine and Pharmacy, 700115 Iasi, Romania

**Keywords:** Artificial Intelligence, diagnostic advancements, UTIs, urinary infection management, Urolithiasis

## Abstract

Urinary tract infections (UTIs) are a common pathology worldwide, frequently associated with kidney stones. We aimed to determine how artificial intelligence (AI) could assist and enhance human medical activities in this field. We performed a search in PubMed using different sets of keywords. When using the keywords “AI, artificial intelligence, urinary tract infections, *Escherichia coli* (*E. coli*)”, we identified 16 papers, 12 of which fulfilled our research criteria. When using the keywords “urolithiasis, AI, artificial intelligence”, we identified 72 results, 30 of which were suitable for analysis. We identified that AI/machine learning can be used to detect Gram-negative bacilli involved in UTIs in a fast and accurate way and to detect antibiotic-resistant genes in *E. coli*. The most frequent AI applications for urolithiasis can be summarized into three categories: The first category relates to patient follow-up, trying to improve physical and medical conditions after specific urologic surgical procedures. The second refers to urinary stone disease (USD), focused on stone evaluation, using different AI and machine learning systems, regarding the stone’s composition in terms of uric acid, its dimensions, its volume, and its speed of detection. The third category comprises the comparison of the ChatGPT-4, Bing AI, Grok, Claude, and Perplexity chatbots in different applications for urolithiasis. ChatGPT-4 has received the most positive evaluations. In conclusion, the impressive number of papers published on different applications of AI in UTIs and urology suggest that machine learning will be exploited effectively in the near future to optimize patient follow-up, diagnosis, and treatment.

## 1. Introduction

*UTI Burden, Prevalence, Guidelines, and Antimicrobial Resistance*

The European Centre for Disease Prevention and Control (ECDC) estimated that 4% of intensive care unit (ICU) patients staying in hospital for over two days had UTIs in 2021. The five most frequently isolated microorganisms in ICU-acquired UTI episodes, by country/network, EU/EEA, 2021, included *E. coli*, *Enterococcus* spp., *Pseudomonas aeruginosa, Klebsiella* spp., and *Proteus* spp. Antimicrobial treatment consisted of 50% empirical therapy days, 38.0% directed therapy days, and 9% prophylactic therapy days [1].

Researchers from ReAct, Uppsala University in Sweden, along with the European Society of Clinical Microbiology and Infectious Diseases Study Group for Antimicrobial Stewardship (ESGAP), designed a study following survey reporting guidelines to map national UTIs guidelines. These authors evaluated the recommendations for antibiotic treatment, factors considered important when setting the guidelines, procedures for evidence grading (if provided), and the availability of resistance surveillance data. There was significant variation in UTI antibiotic treatment recommendations across 15 European countries. Their comparison of existing national guidelines could be a starting point for identifying knowledge gaps and opportunities for improvement in the management of UTIs. Importantly, the availability of antibiotics in each country should be based on clinical evidence and medical needs, rather than financial considerations. Given the rapid increase in antimicrobial resistance among uropathogens and the limited development of new antibiotics, addressing this issue should be considered a high priority [2].

The existence of 18 carbapenemase variants, primarily within ST131 clades A and C, was discovered through the analysis of 594 isolates of *E. coli* sequence type (ST)131 and its single-locus variations containing carbapenemase genes from 17 European Union/European Economic Area countries. An additional warning sign of the worsening epidemiological status of carbapenemase-producing *Enterobacteriaceae* (CPE) in the EU/EEA is the more frequent detection of *E. coli* ST131 carrying carbapenemase genes, which may promote community transmission and dissemination. Carbapenems could become useless for the empirical treatment of severe *E. coli* infections if *E. coli* carbapenemase genes continue to spread. To improve the management of CPE in the EU/EEA and around the world, immediate public health intervention is required [3].

UTIs present a significant burden for the pregnant population. In a study performed in a Gynecology and Obstetrics Hospital from Romania that analyzed the cases of 371 pregnant patients with UTIs, the authors found *E. coli*, *Enterococcus faecalis*, and *Klebsiella* species to be most commonly involved in multidrug-resistant (MDR) infections. An increased risk of preterm birth, premature rupture of membranes, neonatal respiratory distress syndrome, and neonatal intensive care unit admission was detected in patients with MDR infections [4].

In recent years, many medical fields have tried to implement artificial intelligence (AI) in optimizing the diagnosis of different diseases. For example, in 2023, Smith LA et al. from Australia presented their results in a systematic review and meta-analysis regarding AI involvement in patients with chronic obstructive pulmonary disease. The authors compared the performance of machine learning (ML) and deep learning (DL) prognostic models and identified pathways for future research. The team found limited evidence that traditional ML and DL prognostic models outperform conventional disease severity scores [5].

In a recent *Lancet* review from September 2024, Ho CS et al. underlined the potential roles of AI in limiting the antimicrobial resistance (AMR) phenomenon. It is known that AMR causes many issues, given the high prevalence of multidrug-resistant bacterial strains, such as extended-spectrum beta-lactamase (ESBL)-positive *E. coli,* methicillin-resistant *Staphylococcus aureus* (MRSA), *Streptococcus pneumoniae, E. faecium,* and *Clostridioides difficille* [6]. With advancements in AI technology, computational power, and big data, AI is becoming a powerful tool in combating infectious diseases and managing antimicrobial resistance. This includes antibiotic testing, resistance surveillance, stewardship, diagnostics, and the development of antimicrobial drugs. AI may analyze vast datasets utilizing computers and machines, employing either manual feature engineering (i.e., ML) or representation learning, such as deep neural networks analogous to human cognition (i.e., deep learning), to produce new knowledge (i.e., generative artificial intelligence) [7].

It is important to emphasize the cross-domain success of AI. Another medical field which assessed AI as a potential diagnosis tool is radiology. AI-based medical decision systems are being developed and commercialized at a rate that far exceeds our understanding of their utility for physicians. Radiologists emphasize that future research on clinician–AI interaction could improve the cognitive aspects of decision-making and boost the safety and usefulness of AI models in high-risk medical scenarios [8]. Moreover, in a recent paper, Ouyang. D. et al. mentioned the need for more randomized clinical trials focusing on AI, as they found no difference in diagnostic accuracy between AI assistance and standard-of-care assessment in stress echocardiography [9]. Dippel J. et al. examined histological pictures from gastrointestinal samples including numerous malignancies. The authors suggested a deep anomaly detection method that can identify all less common diseases with just training data from common diseases. Pathologists can benefit greatly from this anomaly detection algorithm’s ability to spot unusual cases, improve case prioritization, and reduce missed diagnoses. It could encourage AI use and automation in regular diagnostics and beyond by making AI models safer in histopathology [10].

Porsdam Mann S. et al. presented and analyzed the Artificial Intelligence Act’s key implications for physicians and medical innovators in the United States. The authors presented the potential to influence global medical AI policy and mentioned that U.S. healthcare stakeholders must engage proactively with this new regulatory framework to remain competitive and gain access to the EU market [11].

UTIs represent a medical condition frequently identified in hospitals. Bilsen MP et al., 2024, in a multidisciplinary Delphi consensus study, provided a reference standard for UTI research. The major symptoms for indicative UTI, including newly onset dysuria, urinary frequency, and urinary urgency, plus pyuria, were incorporated into this reference standard. An important change was that the researchers lowered the traditional bacteriuria threshold (from 10^5^ CFU/mL to 10^4^ CFU/mL). It was advised that UTI research be conducted over a wide range of patient demographics using this updated reference standard, which may improve study uniformity. This reference standard can optimize the internal and external validity of diagnostic and therapeutic studies for a disease (e.g., UTI) that imposes a substantial burden on patients and society, particularly in an era of increasing antimicrobial resistance [12].

**Aim:** AI is utilized in various medical fields, as evidenced by the cited papers. This article analyzes AI’s role in detecting UTIs’ etiologic agents and its potential in managing urolithiasis to enhance early diagnosis performance without compromising sensitivity and specificity, ultimately improving patients’ quality of life.

We performed a search in PubMed using different sets of keywords. When using the keywords “AI, artificial intelligence, urinary tract infections, *Escherichia coli* (*E. coli*)”, we identified 16 papers, from which 12 fulfilled our research criteria. When using the keywords “urolithiasis, AI, artificial intelligence”, we identified 72 results, from which 30 were suitable for analysis. Inclusion criteria: articles in English, published between January 2020 and January 2025 (for UTIs) and between January 2024 and January 2025 (for USD), and discussing the application of AI in UTIs and USD. Exclusion criteria: non-English articles, publications without full-text access, peer-reviewed journal articles, conference papers, and studies not specifically focused on AI in UTIs and USD.

## 2. AI and the Detection of Bacterial Strains Involved in UTIs

It is widely accepted that *E. coli* is the most common etiologic agent of UTIs, followed by *K. pneumoniae, P. aeruginosa, Streptococcus agalactiae, E. faecalis,* and *Staphylococcus saprophyticus.* We aimed to determine whether any AI systems have been developed for the assessment of UTIs as part of advancements in diagnostic technologies.

We conducted an analysis of the PubMed database using the keywords “AI, artificial intelligence, UTIs, *E. coli*” to find papers published over the past five years. Our search identified 16 papers, of which 12 met our inclusion criteria outlined by the keywords (see Table 1).

It is important to mention that AI UTI assessment tools have been developed all over the world (e.g., the USA, Korea, India, Poland, Italy, Israel, Taiwan, China, etc.) for the assessment and fast and accurate detection of etiological agents, in the prevention of infection (e.g., special catheter development), for identifying genes related to resistance to antibiotics and to simultaneously evaluate etiology, and for the prediction of antibiotic treatment efficiency.

Identification of bacterial agents: The developed AI models produced different results, with different clinical utilities—they were able to predict UTI analysis for a large variety of microorganisms (Gram-positive and Gram-negative bacteria and fungi) and predicted that the elderly had a higher risk for developing invasive *E. coli* diseases. One system used deep neural networks and large-volume microscopy to accurately detect uropathogenic *E. coli.* Another used a geometric design optimized by an AI model for the faster detection of this Gram-negative bacillus.

Resistance gene detection: A research group developed an artificial neural network (ANN) for predicting the clinical efficacy of empirical antimicrobial treatment in women with recurrent UTIs, and in their view it seemed that this system was able to guide the antimicrobial choice in the management of recurrent UTIs at the point of care. In addition, one AI system was scientifically and biologically validated to detect antibiotic resistance genes in *E. coli.* While these groups analyzed bacterial etiology and antibiotic resistance separately using AI, their combined analysis was published in another study, comprising bacterial etiology identification and the assessment of susceptibility to antibiotics using infrared microscopy. This analysis was performed using ML (machine learning), taking a maximum of 40 min after receiving the patient’s urine sample. Another paper combined the whole-genome sequencing of pre- and post-treatment bacterial isolates with the ML analysis of UTIs. The authors found that resistance-gaining recurrences can be predicted using the patient’s past infection history and minimized by means of machine learning-personalized antibiotic recommendations.

**Figure 1:**
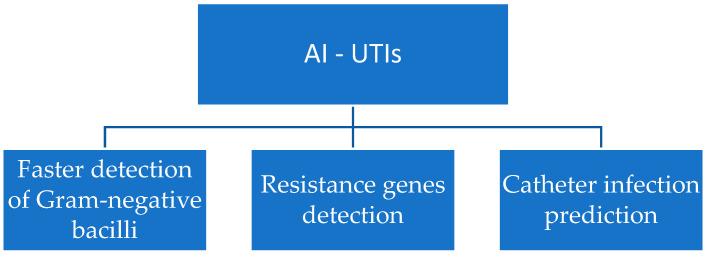
The benefits of using AI for the improved diagnosis of UTIs.

Catheter infection prediction: An important risk factor in the development of UTIs is urinary catheters, which are required in various medical specialties. In a recent study which investigated the occurrence of carbapenem-resistant (CR) *K. pneumoniae* infections in patients admitted to a urology center, the presence of urinary catheters inserted for up to one month was found to be a risk factor for such infections [13]. Another study on a similar topic found significant correlations between urinary catheterization and older age and with the presence of an isolate with extensive drug resistance (XDR) or pan-drug resistance (PDR), which are very well known as risk factors for a high mortality rate. The antimicrobial susceptibility of the tested strains was lower for catheterized patients, especially for carbapenems and aminoglycosides [14]. In the context of multidrug-resistant isolates, especially in countries where their prevalence is high (e.g., Greece, Romania, and Spain), a group of researchers proposed a novel geometric design, optimized by an artificial intelligence model, based on the physical mechanism of upstream swimming to improve the suppression of bacterial contamination at the upstream end (Table 1, [15,16,17,18,19,20,21,22,23,24,25], Figure 1).

AI in the identification of UTIs related to *P. aeruginosa: P. aeruginosa* was also studied as an etiological agent of UTIs in connection with AI. This bacterium does not occur as an etiological agent of UTIs as frequently as *E. coli*, but it is recognized as being a difficult-to-treat bacterium because of its natural resistance to many antibiotics. In a study which also analyzed *E. coli*, the authors developed an AI system which had good performance in predicting urine cultures positive for *P. aeruginosa* [15]. Using infrared spectroscopy, Abu-Aqil G et al. created an artificial intelligence method that reduces the time required to identify and test for antibiotic susceptibility in *Proteus mirabilis* and *P. aeruginosa* from 48 h to about 40 min, directly from patient urine samples. The authors of this article claimed to have identified *P. aeruginosa* with 99% accuracy while also determining the isolates’ susceptibility to various antibiotics with an accuracy of over 80% [26].

The outlook for all the above clinical applications of AI is optimistic, showing promise for advancing patient education, risk stratification, disease diagnosis, and personalized treatment strategies. However, there is a need for the follow-up classical medical diagnosis of bacterial infections and of resistance gene detection.

From 2018 to 2023, generative models improved nephrology by boosting diagnostic precision, patient education, and clinical workflows. Tools like ChatGPT-4 aided chronic kidney disease dietary guidance and education, though human oversight remained necessary due to depth and citation issues. By 2024, advanced generative and multimodal models further enhanced diagnosis, prediction, education, and clinical support in nephrology. Generative models like ChatGPT-4 and Sora have been explored for medical education, diagnosis, and personalized guidance in renal medicine. GPT-4 is trained to identify relationships between textual and visual data, allowing it to generate text responses that incorporate information from both modalities. SORA (2024) is a text-to-video generation model developed by OpenAI. It employs a transformer-based architecture integrated with a pre-trained diffusion model to produce videos from textual descriptions [27].

Like any new tool, even AI models should be clinically validated. Neha F et al. also presents the limitations of new technologies, such as knowledge cutoffs, a lack of contextual understanding, challenges, data representation biases, computational demands, and ethical concerns [27].

**Table 1:** AI systems focused on *E. coli*-related UTIs.

No.	Author, Year, Country	AI Model	Results	Medical Importance of AI System
1	Choi MH et al., 2024, South Korea [15].	Predictive AI urine analysis model 360,376 patients suspected of having UTIs.	AUROC of 0.872 good performance for: Gram-negative bacteriuria (0.901); Gram-positive bacteriuria (0.745), Funguria (0.872).	Improved performance for detecting: - Gram-positive, negative bacteria, - fungi.
2	Clarke E et al., 2024, USA [16].	XGBoost model 663 distinct patients.	XGBoost model had an average precision of 0.0031	The feasibility of databases and ML to risk factors for infectious diseases, including invasive *E. coli* disease.
3	Nayak DSK et al., 2024, India [17].	aiGeneR designed to identify antibiotic-resistant genes in *E. coli*.	aiGeneR identified the tetM resistance gene accuracy of 93%, AUC of 0.99 (*p* < 0.05).	Scientifically and biologically validated to successfully detect *E. coli* resistance genes.
4	Iriya R et al., 2024, USA [18].	Deep learning algorithms to facilitate culture-free bacterial detection. low magnification to visualize adequate sample volumes.	Superior accuracy in detecting uropathogenic *E. coli* compared to traditional ML methods. higher validation accuracy (75% for *E. coli*, 82% for urine particles).	Rapid and efficient alternative to traditional culture-based methods.
5	Zhou T et al., 2024, USA [19].	Microfluidic experiments anti-infection mechanisms of the new AI-designed catheters.	AI-aided geometric design of the catheters significantly improved the suppression of bacterial contamination at the upstream end.	The effectiveness of the AI-driven geometric design of anti-infection catheters: integrating microfluidic testing and 3D printing: development of safer and more effective urinary catheters.
6	Wityk P et al., 2023, Poland [20].	Absorption spectroscopy supported by ML 86 individuals with proven *E. coli*-induced UTIs *E. coli*-induced urosepsis (155 isolates).	97% accuracy for raw data no additional measurements to normalize the dataset.	Sensor simplicity, portability, versatility, and cost-effectiveness.
7	Cai T et al., 2023, Italy [21].	ANN for predicting the clinical efficacy of the empiric antimicrobial treatment 1043 female patients 725 cases were used for training 318 for validation.	Sensitivity of 87.8% specificity of 97.3% in predicting the efficacy of empirical therapy *E. coli* resistance to cotrimoxazole and amoxicillin-clavulanic acid was a significant predictor of failure in fosfomycin-based treatments.	Directing empirical antibiotic treatment at the point of care for patients with recurrent UTIs.
8	Abu-Aqil G et al., 2022, Israel [22].	Fourier-transform infrared (FTIR) spectroscopy combined with ML techniques. 1765 different UTI cases.	Accuracy of approximately 96% (compared to MALDI-TOF results) antibiotic susceptibility determination accuracy was about 84% (compared to the Vitek 2 system results).	Reduce the time required for susceptibility testing from days to about 40 min good accuracy profile compared to gold-standard methods.
9	Jeng SL et al., 2022, Taiwan [23].	3 machine learning models were developed: logistic regression (LR), decision tree (DT), and random forest (RF) 963 patients.	Prediction accuracy was higher (0.700, 0.604, and 0.654 during the initial clinical visit; 0.709, 0.604, and 0.635 following hospitalization, respectively).	Greater accuracy in anticipating the appearance of a single uropathogen (*E. coli*) in recurrent UTIs.
10	Dong F et al., 2022, China [24].	AI algorithms to identify potential pathogens and other relevant biomarkers associated with UTIs. 146 urine samples.	The AI-based method showed: sensitivity: 69.5%; specificity: 96.5% positive predictive value: 93.2% negative predictive value: 82%.	Sufficient specificity for UTIs, reduce unnecessary urine cultures of negative samples.
11	Stracy M et al., 2022, Israel [25].	ML system that combined whole-genome sequencing 1113 bacterial isolates before and after treatment with a dataset analysis of 140,349 UTIs.	Identifications of patterns and predictors of treatment-induced resistance.	Machine learning-based personalized antibiotic recommendations could provide a mitigation strategy to diminish the emergence and dissemination of resistant pathogens.

## 3. AI in the Analysis and Management of UTIs, Urolithiasis, and Stones

Different research teams are trying to test and validate artificial intelligence and ML applications in the clinical practice of urology. By analyzing increasingly extensive datasets, researchers are trying to detect patterns that could facilitate patient preselection. Moreover, efforts are underway to reveal previously unknown associations. Initiatives are underway in urology to enhance the management of UTIs and kidney stones, utilizing interconnected computer systems. Big data and artificial intelligence are expected to bring novel developments in the coming years [28].

Campobasso D et al. conducted a scoping review to elucidate the present utilization of AI in predicting infectious complications in patients with urolithiasis. They conducted an analysis of retrospective data published between 2021 and 2023, employing several AI algorithms. Random forest and artificial neural networks provide superior specificity and sensitivity, outperforming standard statistical analyses. However, these systems must acknowledge external validation to enhance the selection of variables for analysis by AI models in predicting urosepsis [29].

In a randomized controlled trial, Hong X et al. developed a predictive model for urosepsis risk in patients with upper urinary tract calculi. They employed an artificial neural network to analyze variables such as gender, age, body temperature, diabetes history, urine leukocytes, urine nitrite, urine glucose, and the extent of hydronephrosis. Their validation and training yielded reliable values for sensitivity, specificity, and AUC, leading the authors to conclude that their AI model is applicable for risk assessments of urosepsis in patients with lithiasis [30].

**Figure 2:**
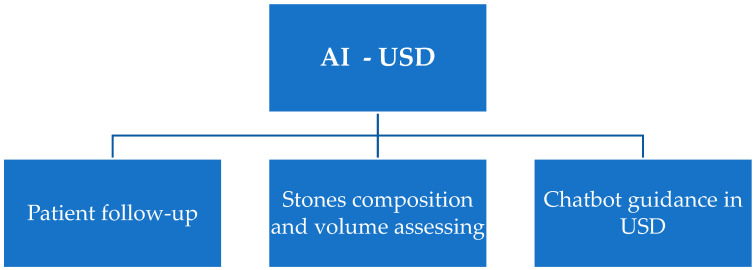
The benefits of using AI for USD.

Starting from such promising examples, we conducted a search in the PubMed database utilizing the phrases “urolithiasis, AI, artificial intelligence,” and from the 72 results obtained, we selected 30, which are presented in Table 2. The search was conducted for papers published in the previous year (January 2024–2025). We limited our search to one year, as the five-year filter provided by PubMed yielded an excessive number of papers for analysis in this study (197 results). The chosen 30 papers originate from every continent. Table 2 included six papers from China, five from the USA, and three from the UK, along with contributions from Canada, India, Italy, Taiwan, Iran, Turkey, and France. The predominant AI applications for urolithiasis can be categorized into three groups: The first pertains to patient monitoring, aimed at enhancing physical and medical conditions post-surgical interventions (e.g., retrograde intrarenal surgery) by identifying individuals at risk for febrile UTIs or elevated susceptibility to sepsis. These studies examined adult patients and the pediatric population. The rapidity and precision of AI deployment were regarded as essential instruments for reducing the documentation load and enhancing patient–clinician relations. The second direction pertains to the assessment of stones, employing various AI and machine learning methods to analyze the stone’s composition in terms of uric acid, as well as the stones’ size, volume, and detection speed. The third group of articles aim to compare various chatbots based on their capacity to provide prompt and accurate responses to the posed inquiries. These chatbots include ChatGPT-4, Bing AI, Grok, Claude, and Perplexity, and they are considered under various conditions, including guidelines for surgical stone management, juxtaposed with EAU guideline recommendations. GPT-4 exhibited the most complex linguistic structure in all comparative investigations. There is a need for a deep analysis of the clinical implications of chatbot comparison, as they have been used in very non-homogenous applications. It is important to understand and validate the way in which the linguistic complexity or reference presence can impact clinical decision-making (Table 2, [31,32,33,34,35,36,37,38,39,40,41,42,43,44,45,46,47,48,49,50,51,52,53,54,55,56,57,58,59], Figure 2).

Many recent explainable AI reviews in medical imaging have presented the limitations of AI models, such as the need for integrating more diverse and high-quality data to enhance the global effectiveness of AI models in melanoma diagnoses. Authors have recommended standardizing the databases and developing more robust and explainable models to guide future research [60]. According to Peck M et al., explainable AI solutions are still required in order to win over clinicians and promote wider usage [61]. Another research team pointed out important research gaps and suggested ways to create trustworthy, effective AI models that incorporate explainable AI techniques to revolutionize the diagnosis of breast cancer [62]. The studies analyzed are heterogenous regarding their sample size and validation methods, and this raises questions regarding real-world clinical implementation challenges.

## 4. European Guidelines Regarding UTIs

Recent guidelines authored by German researchers examined whether antibiotic prescriptions had evolved over time in alignment with established protocols. To try and reduce antibiotic resistance and adverse effects, these guidelines advocate for fosfomycin, nitrofurantoin, pivmecillinam, and nitroxoline as first-line therapies, while advising against the use of broad-spectrum antibiotics, including fluoroquinolones and cephalosporins. The authors conducted a retrospective analysis of 1.7 million UTI prescription cases utilizing routine data from 2013 to 2019. In females, the proportion of fluoroquinolone prescriptions declined significantly with time, while the proportion of the first-line agents fosfomycin and pivmecillinam rose. Gynecologists prescribed the largest proportion of first-line medication in comparison to general practitioners and urologists. Fluoroquinolone use declined across all three specialism categories. Fosfomycin prescription percentages declined, whilst those of nitrofurantoin, nitroxoline, and cephalosporins grew. The authors’ findings indicate a trend towards increased adherence to guidelines in UTI therapy, marked by a considerable rise in the use of fosfomycin and pivmecillinam, particularly among women, alongside a notable decline in the use of fluoroquinolones [63].

The Task and Finish Group for Urinalysis and the European Federation of Clinical Chemistry and Laboratory Medicine have revised the European Confederation of Laboratory Medicine (ECLM) European Urinalysis Guidelines (2000) regarding urinalysis and urine bacterial culture, aiming to enhance the precision of these assessments in European clinical laboratories and to assist the diagnostic industry in the development of new technologies. Kouri TT et al. conducted an analysis of specimen collection, emphasizing contamination rates (cultures) and urine density (chemistry, particles). These authors advocate for the use of chromogenic agar as the major medium in urine cultures and support the use of leukocyturia to minimize less critical antimicrobial susceptibility tests. In recent years, automation in bacteriology has been advocated to reduce turn-around times, and matrix-assisted laser desorption ionization time-of-flight mass spectrometry (MALDI-TOF) is suitable for the swift identification of uropathogens, both familiar and new ones, such as *Aerococcus urinae, A. sanguinicola*, and *Actinotignum schaalii* [64].

Recent guidelines from the European Association of Urology (EAU) and a clinical prediction rule established by Van Nieuwkoop et al. propose straightforward criteria for conducting radiological imaging in patients with febrile UTIs. Vanolli K. et al. examined the medical records of individuals with a UTI from four hospitals in Switzerland, taking into account clinically relevant imaging abnormalities correlated with a history of urolithiasis, extensive hematuria, and known urogenital malformations. Consequently, compliance with imaging criteria was inadequate, particularly when this investigation was not advised. Nonetheless, supplementary elements linked to clinically relevant outcomes indicate the need for a novel, effective clinical prediction rule [65].

## 5. American Guidelines Regarding UTIs

UTIs are common in children. Cystitis is a bladder infection or inflammation, while pyelonephritis affects the kidneys. Pyelonephritis can cause renal scarring, which may lead to nephrosclerosis, hypertension, and chronic renal failure. The purpose of imaging is to direct treatment by identifying patients at elevated risk for recurrent UTIs or renal scarring. The Expert Panel on Pediatric Imaging offers preliminary imaging guidelines for children exhibiting their first febrile UTIs with a suitable medical management response, atypical or recurring febrile UTIs, and subsequent imaging for children diagnosed with vesicoureteral reflux. The American College of Radiology Appropriateness Criteria are standards for specific clinical scenarios based on evidence and reviewed annually by a team of experts from various disciplines. Guideline preparation and modification enable the systematic evaluation of the medical literature from peer-reviewed publications, serving as valuable tools for clinicians worldwide [66].

An illustrative example is the study from 2024 of Khoury L. et al., which revised the 2011 American Academy of Pediatrics (AAP) guidelines for diagnosing UTIs in infants aged 2–24 months following an analysis of 1432 patients under 6 months of age who underwent urine culture tests throughout the research period. The novel diagnostic technique (guidelines II) applies to patients over 2 months old, requiring an abnormal urine test and a lower colony count threshold for diagnosis compared to the AAP guidelines [67].

## 6. UTIs, Antimicrobial Resistance, Antimicrobial Stewardship, and Clinical Trials

From 2020 to 2025, eight clinical trials on UTI and antimicrobial stewardship were published. To cover this topic thoroughly, we briefly present the most representative examples. Gohil SK et al. conducted a randomized controlled trial to determine whether computerized provider order entry (CPOE) prompts that provide MDRO (multidrug-resistant organism) risk estimations unique to patients and pathogens could decrease the usage of empirical extended-spectrum antibiotics for treating UTIs. Despite the low chances of patients contracting MDRO infections, doctors often overuse extended-spectrum antibiotics. Safe measures to reduce the overuse of empirical antibiotics are needed. The authors studied the effects of routine stewardship in 30 hospitals and a CPOE stewardship bundle on antibiotic selection during the first three hospital days. The group that used CPOE prompts experienced a 17.4% decrease in empirical extended-spectrum treatment days as compared to routine stewardship. CPOE prompts with real-time recommendations for standard-spectrum antibiotics reduced the use of extended-spectrum antibiotics in non-critically ill UTI patients with low MDRO risk compared to standard stewardship [68].

König E. et al. from Austria conducted a non-randomized trial to increase appropriate antibiotic treatments for UTIs in long-term care institutions using a multifaceted antimicrobial stewardship intervention. The intervention’s core elements included onsite training for nursing staff, the provision of written guidelines, development of the project webpage to disseminate guidelines and films, and voluntary continuing medical education for primary care physicians. A total of 326 UTI events were documented, with 165 occurring in the control group and 161 in the therapeutic group. The proportion of correct antibiotic selections in the intervention group increased by 8.9% overall, from 42.1% prior to the intervention to 45.9% during the intervention and reaching 51% post-intervention. The percentage was lower in the control group, and there were no appreciable differences between the intervention and control groups in terms of the safety outcomes (the percentage of clinical failure, the number of hospitalizations for UTIs, and adverse events brought on by antimicrobial treatment). During the intervention period, there was a notable increase in appropriate treatments (in terms of choosing to treat the UTI) thanks to an antimicrobial stewardship program that included practice standards and local and online education for general practitioners and nursing staff. The post-intervention phase did not maintain this difference. The cited study concluded that ongoing efforts are essential to significantly improve prescription quality [69].

In a randomized, double-blind, controlled phase III noninferiority clinical trial, Butler DSC et al. compared BNO 1045 with fosfomycin for the treatment of uncomplicated UTIs. The scientists found that the two therapies changed urine cytokine levels. Novel therapies are of vital importance considering the rise of AMR, and it has been demonstrated that the phytotherapeutic medication BNO 1045 (Canephron^®^ N) is not inferior to conventional antimicrobial management. Women with uncomplicated UTIs who were treated with BNO 1045 or fosfomycin had their urine samples examined for IL-6 and IL-8 levels using analyte-to-creatinine ratios. Both treatments reduced urine IL-6 and IL-8 levels in a manner comparable to that of fosfomycin therapy. According to the authors of this clinical study from the USA and Germany, BNO 1045 is similar to fosfomycin treatment in lowering the local inflammatory response linked to uncomplicated UTI in addition to symptom reduction [70].

A pragmatic, parallel, cluster-randomized controlled study conducted by Hartman EAR et al. examined whether a multimodal antibiotic stewardship intervention could reduce the prescription of antibiotics for suspected UTIs in frail older individuals. This study, which covered 1041 fragile older persons aged 70 or older, was conducted in four countries (Poland, the Netherlands, Norway, and Sweden) between September 2019 and June 2021. Healthcare workers were given a comprehensive antibiotic stewardship intervention that included a decision-making tool for the proper use of antibiotics and a toolbox filled with instructional resources. The primary outcome was the annual number of antibiotic prescriptions for suspected UTIs per person. The intervention group had a lower likelihood of being prescribed antibiotics for a suspected UTI diagnosis compared to the usual care group. This complex antibiotic stewardship intervention effectively reduced antibiotic prescriptions for suspected UTIs in older individuals without compromising safety [71].

Two pragmatic trials examined the impact of quarterly audits and feedback on antibiotic prescribing among primary care physicians for respiratory tract infections, UTI management, and community antibiotic resistance. The trials focused on reducing antibiotic prescriptions through a primary-care-provider-focused antimicrobial stewardship intervention (Aghlmandi S et al. from Switzerland and McIsaac W. et al., from Toronto, Canada). The results of these trials varied: In Canada, the intervention reduced antibiotic prescriptions for respiratory and urinary infections, increased delayed prescriptions, and shortened prescription durations. However, it had no effect on primary care physicians in Switzerland with medium to high antibiotic prescription rates [72,73]. Nace DA et al. conducted another randomized controlled trial in the United States. Antimicrobial use among residents with unlikely cystitis was the focus of a quality improvement intervention study. The incidence of antibiotic treatment for instances with improbable cystitis, as determined by published criteria, was the main result. Compared to control institutions, intervention facilities treated fewer improbable episodes of cystitis with antibiotics during the intervention. According to this study, in a cohort of nursing homes, a low-intensity, comprehensive intervention was linked to better antibiotic prescribing for uncomplicated cystitis without having a negative correlation with other safety outcomes [74].

The ESCMID general competencies in antimicrobial prescribing and stewardship cover microbiology, pathophysiology, infection diagnosis, antimicrobial prescribing, and stewardship. Broad-spectrum antimicrobials should not be used unnecessarily [75]. Proactive auditing and feedback, self-directed antibiotic reassessments by prescribing clinicians, antibiotic dose optimization, and antibiotic duration are examples of antimicrobial stewardship interventions that should be implemented before or at the time of prescription (e.g., clinician, patient, and public education, institution-specific guidelines for the management of common infections, etc.). These antimicrobial stewardship efforts may include AI after thorough research [76].

Up to three days before urine antibiotic susceptibility test availability, AI systems trained on a sizable dataset may accurately predict urine culture susceptibility vs. resistance. Using such an algorithm in clinical settings could enhance antimicrobial stewardship and clinical care [77].

## 7. Conclusions

UTIs are a significant pathology, evidenced by the numerous recent papers on diagnosis guidelines and antimicrobial stewardship to combat resistance. This manuscript presents a timely review of the applications of AI in the diagnosis and management of UTIs and urolithiasis.

AI can help to detect UTI causes, especially for Gram-negative and some Gram-positive bacteria. The future use of whole-genome sequencing may identify new bacteria playing key roles in UTI etiology.

In urology, AI could be implemented in patient follow-up after surgery, in detecting urinary stones quickly, and in analyzing their composition and volume. ChatGPT-4 received high evaluations from many urologists for the quality of its medical information. AI and ML should follow current medical guidelines for UTI diagnosis to prevent MDR bacterial spread, promote antibiotic stewardship, and optimize urological surgeries.

All of the above approaches to utilizing AI should be analyzed in further research, including prospective validation, to address their main limitations, including knowledge cutoff, difficulties in contextual understanding, bias and demographic representation issues, and ethical challenges.

## Figures and Tables

**Table 2:** AI applications in urolithiasis.

No.	Author, Year, Country	Aim of Study	AI Method Urolithiasis Results	Clinical Importance
PATIENT FOLLOW-UP				
1.	Senel C et al., 2024, Turkey [31].	This research team aimed to create a new scoring system and determine risk factors regarding febrile UTIs using machine learning methods for patients who underwent retrograde intrarenal surgery (RIRS).	The study included 511 patients who underwent RIRS, 34 of whom developed febrile UTI. Hydronephrosis, a history of post-ureterorenoscopy UTI, and urine leukocyte count were identified as significant independent predictors of febrile UTI after RIRS. With at least one of these characteristics, 32 out of 34 patients (94.1%) who had a postoperative F-UTI were accurately predicted.	This novel scoring system formulated based on three factors, hydronephrosis, previous post-ureterorenoscopy UTI, and urine leukocyte count, can effectively differentiate patients at risk for the development of UTI following RIRS.
2.	Castellani D et al., 2024, Italy [32].	A machine learning model was developed to predict the risk of sepsis in patients who underwent RIRS.	Data from 1552 patients who underwent RIRS in 15 centers was processed using random forest, decision tree and gradient boosting algorithms.	The web-based interface of the predictive model was made available by the authors at https://emabal.pythonanywhere.com/. (accessed on 6 June 2025) It can predict post-RIRS sepsis with high accuracy and may be used for patient selection for day-surgery procedures and for identifying patients at higher risk of sepsis.
3.	Chang YJ et al., 2025, Taiwan [33].	This research introduces a biosensor for measuring uric acid concentration in calculi and a deep learning-based ANN system for assessing chronic kidney disease (CKD) risk.	The ANN system included age and creatinine values as inputs. A screen-printed electrode chip was used to measure uric acid concentration by cyclic voltammetry. The uric acid concentration in stones was measured using the biosensor, and the result was translated into serum uric acid concentration, facilitating the estimation of creatinine levels, which were subsequently utilized by the ANN to evaluate the risk of developing CKD.	This system can assist urologists in determining whether patients should seek consultation with nephrologists for early diagnosis and treatment.
4.	Geraghty RM et al., 2024, UK [34].	To build, optimize, and validate a machine learning model for predicting percutaneous nephrolithotomy (PCNL) outcomes using a national database.	Data from 12,810 patients in a prospective national database was used to build extreme gradient boosting, deep neural network, and logistic regression models for each outcome of interest (intensive care admission, postoperative infection, transfusion, adjuvant treatment, postoperative complications, visceral injury, and stone-free status at follow-up) in complete cases.	This machine learning study on PCNL provides a tool that could be used by surgeons to predict this procedure’s outcome and advise the patients about the risk of complications and the outcomes they can expect.
5.	Moryousef J et al., 2024, Canada [35].	To assess the integration of artificial intelligence (AI) scribing technology within the field of urology as a potential solution to streamline documentation processes and enhance clinical efficiency.	Standardized reference consultation notes for common urologic referrals, including urolithiasis, benign prostatic hyperplasia, and prostate-specific antigen screening, were developed. Audio recordings of simulated patient interactions were presented to five publicly available AI scribes. The authors report that the AI system demonstrated high accuracy in transcribing conversations and capturing relevant clinical information. Of the evaluated AI technologies, Nabla achieved the highest efficacy, attaining a composite score of 68% and the lowest critical error composite score of 28%.	AI scribes can significantly lessen the administrative load of clinical recording, thus mitigating burnout, but as a complementary tool, with the clinician still being responsible for maintaining the quality of patient care.
6.	Nedbal C et al., 2024, UK [36].	To evaluate the effectiveness of ML in predicting outcomes of flexible ureteroscopy with laser lithotripsy for kidney stone disease, on the basis of preoperative characteristics.	Data from a large endourology center was used to develop and validate ML models for predicting clinical outcomes. The results indicate that the ML frameworks demonstrate high accuracy (93%) and precision (87%) in predicting stone-free status, as well as complications such as hydronephrosis and septic events associated with the stone removal procedure. The majority of complications were linked to a positive urine culture prior to surgery.	The final model was over 90% accurate in predicting complications and stone-free status post-surgery. These technological advances could assist urologists in overcoming the traditional limitations of ureteroscopy.
7.	Vigneswaran G et l., 2024, UK [37].	To create a model that predicts stone-free status after ureteroscopy by analyzing stone volume with additional clinical and radiographic parameters.	The ML model was trained to analyze the relationship between stone volume and the likelihood of achieving a stone-free status post-procedure. The accuracy and AUC of a fivefold cross-validated RUS-boosted tree model were 74.5% and 0.82, respectively, while the sensitivity and specificity were 75% and 72.2%, respectively.	It is possible to anticipate which patients will be stone-free after ureteroscopy using machine learning. Total stone volume seems to be more significant than stone size as a predicting factor.
8.	Meng X et al., 2024, China [38].	To investigate the efficacy of a novel surgical navigation system that integrates DL and mixed-reality technologies in guiding puncture during PCNL for the minimally invasive removal of kidney stones.	The data of 136 patients with kidney stones was retrospectively analyzed. The study revealed that the use of the navigation system significantly improved the accuracy of the puncture site compared to traditional ultrasound guidance.	Real-time intraoperative navigation with acceptable accuracy and safety is made possible using a navigation system based on DL and mixed reality in PCNL for kidney stones removal.
**STONE ASSESSMENT**				
9.	Cao Y et al., 2024, China [39]	Devising a technique for differentiating pure uric acid kidney stones from non-uric acid stones by analyzing quantitative computed tomography (CT) parameters of single-energy slices of urinary stones in relation to chemical stone classifications.	The team proposed a deep learning framework that leverages convolutional neural networks (CNNs) to automatically classify urinary stones based on their composition from CT images. The dataset included 918 non-enhanced thin-slice single-energy CT images of known chemical stone types, analyzed together with clinical data, and stone composition analysis results. The accuracy of the model was 97.01%, the sensitivity was 84.62%, and the specificity was 82.28%.	This deep learning model offers a rapid diagnostic technique for predicting the uric acid composition of kidney stones, using a CNN analysis of thin-slice single-energy CT images.
10.	Zhu Q et al., 2024, China [40].	To develop a DL model to detect kidney stones early using standard urine and blood test parameters.	Seventeen variables were evaluated and the four most significant characteristics based on the weight coefficient in this model were urine WBC, urine occult blood, qualitative urinary protein, and microcyte percentage. The model demonstrated substantial predictive value for kidney stones. The model achieved an accuracy of 89.5% and an AUC of 0.95.	Routine urine and blood tests can be analyzed using this model to accurately identify the presence of kidney stones, being of assistance to clinicians in the early detection of this condition.
11.	Yenikekaluva A et al., India [41].	To evaluate the UrologiQ AI system, designed to enhance the measurement of kidney stone volume in patients suffering from urolithiasis.	In comparison to radiologists, the AI demonstrated superior accuracy, efficiency, and consistency in quantifying kidney stone volume. The AI measured the volume of kidney stones with an average difference of 80% relative to the volumes determined by radiologists.	By providing reliable and objective measurements of kidney stone volume, the UrologiQ AI system outperforms radiologists’ manual calculations. By integrating AI with kidney stone detection and treatment, there is potential for enhancing diagnostic accuracy and clinical decision-making.
12.	Song R et al., 2024, China [42].	To create a deep learning model utilizing CT images to predict the success of extracorporeal shock wave lithotripsy (ESWL) treatment for patients with ureteral stones above 1 cm in size.	A total of 333 patients who underwent ESWL were allocated into training and test groups. A deep learning model was created to predict ESWL outcomes based on CT calculi images. The model showed significantly better predictive performance in both the training and test groups compared to radiomics.	Analyzing CT scans with this deep learning model could predict the success of ESWL treatment with very good accuracy and could be used as an auxiliary tool in clinical urology.
13.	McMahon AK et al., 2024, USA [43].	To assess and compare the capacity of ChatGPT-4™ (Open AI^®^) and Bing AI™ (Microsoft^®^) in responding to inquiries related to kidney stone treatment, according to the American Urological Association (AUA) guidelines, while evaluating aspects such as suitability, emphasis on consulting healthcare professionals, citations, and compliance with guidelines by each chatbot.	Based on the AUA Surgical Management of Stones guideline, 20 questions regarding kidney stone evaluation and treatment were formulated. These were addressed to the ChatGPT-4 and Bing AI chatbots, and their answers were compared using the brief DISCERN tool as well as response appropriateness. ChatGPT-4 surpassed Bing AI in questions 1–3, assessing clarity, accomplishment, and relevance of responses. Bing AI consistently included references, while ChatGPT-4 did not.	ChatGPT4 outperformed Bing AI in responses with a clear aim, an achieved aim, and in giving relevant and appropriate responses based on AUA surgical stone management guidelines, while Bing AI’s responses could be quality-checked, because it included references.
14.	Mahmoodi F et al., 2024, Iran [44].	To use machine learning in enhancing the identification of people at risk of developing clinically significant kidney stones.	The dataset included 10,128 individuals, for which 102 predictor variables from surveys and tests were analyzed. The presence of symptomatic kidney stones was the chosen outcome variable. Five ML algorithms were applied to examine kidney stone predictors, with performance comparisons made. Data balancing was performed using the synthetic minority over-sampling technique, and the accuracy, precision, sensitivity, specificity, F1 score, and AUC were evaluated for each algorithm.	The primary predictors for kidney stones included serum creatinine, sodium intake, hospitalization history, duration of sleep, and blood urea nitrogen levels. The tested ML models showed potential in evaluating the likelihood of developing systematic kidney stones and could recommend preventive lifestyle changes to reduce the risk.
15.	Liu K et al., 2024, China [45].	Based on CT scans, the authors aimed to create a non-invasive prediction method for identifying kidney stone types.	The authors developed a self-distillation model that uses DL to analyze medical images of urinary stones. By eliminating the need for external teacher models and avoiding the additional computational costs and performance degradation associated with model compression, this technique significantly improves the effectiveness of lightweight models, resulting in a classification accuracy of 74.96% on a private dataset.	These findings further support our model’s viability for clinical implementation, which could help medical practitioners create more accurate treatment strategies and lessen patient discomfort.
16.	Elbedwehy S et al., 2024, Egypt [46].	This paper presents a novel approach to diagnosing kidney diseases (stones, cysts and tumors) through the integration of traditional convolutional neural networks (CNNs).	A hybrid model was created that combines the well-established AlexNet architecture (with robust feature extraction capabilities) with the more recent ConvNeXt framework (with sophisticated attention mechanisms). The dataset included 12,446 CT images that were analyzed by the AI system. The results demonstrate that this hybrid approach significantly enhances classification performance, achieving an accuracy of 99.85% in distinguishing between various kidney disease states.	These findings demonstrate how well the hybrid architecture and optimization approach diagnose renal disorders.
17.	Shee K et al., 2024, USA [47].	To create an effective predictive model that can analyze urine composition data collected over a 24 h period, and to identify patterns that correlate with the likelihood of kidney stone recurrence.	Data regarding 24-h urine samples from patients who had previously experienced kidney stones was analyzed. The results demonstrate a significant correlation between specific urine composition metrics—such as calcium, oxalate, and citrate levels—and the recurrence of stones. The validation set showed moderate discriminative ability in prediction accuracy (AUC = 0.64); repeat modeling with the four highest scoring features showed minimal loss in accuracy (AUC = 0.63).	Stone recurrences can be predicted with moderate accuracy by ML algorithms based on 24 h urine data.
18.	Nedbal C et al., 2024, Italy [48].	Developing an ML predictive model to analyze preoperative characteristics and predict the outcomes of ureteroscopy lasertripsy in a pediatric population.	Fifteen ML algorithms were used to find correlations between preoperative characteristics and postoperative outcomes (stone-free status after 3 months and the appearance of complications). Key predictors identified included factors such as stone size, location, and the presence of anatomical anomalies.	ML has great potential in pediatric urology to significantly aid surgeons in the management of kidney stones.
19.	Kim J et al., 2024, South Korea [49].	The study’s objective was to create an AI system that uses DL to detect urolithiasis in computed tomography (CT) images. This system would be able to calculate stone volume and density in real time, which is crucial for treatment decisions. The system’s performance in emergency scenarios was compared to that of urologists.	For the training of the AI system, 39,433 CT images were used, of which 9.1% were positive. The system’s peak positive-to-negative sample ratio was 1:2, and it had a 95% accuracy rate. Accuracy stayed at 95% in a validation set of 5736 photos, of which 482 were positive.	This AI system can assist in diagnosing urolithiasis with 94% accuracy in actual clinical situations, and even when using standard consumer-grade GPUs, the results could be produced quickly.
20.	Sánchez C et al., 2024, Chile [50].	To develop an ML model that can accurately predict the risk of developing kidney stones, based on patient demographics, medical history, and lifestyle factors.	A prediction model that identified people at risk of kidney stone formation was created, and it had high accuracy (88%). The model utilized different classifiers, such as logistic regression, decision trees, random forests, and extra trees.	This AI-based tool can predict the risk of urinary lithiasis and help clinicians recommend preventive dietary and lifestyle measures.
21.	Leng J et al., 2024, USA [51].	Creating an automated system that can accurately classify kidney stones, based on their composition, using video captured during ureteroscopy.	A dataset of endoscopic videos that included various types of kidney stones was analyzed. The model was trained on labeled data, where the composition of each stone was previously identified by experienced clinicians. The UroSAM model was built to automatically identify kidney stones in the images and recognize the majority stone composition (calcium oxalate monohydrate, dihydrate, calcium phosphate, and uric acid).	This work shows how an ML model can accurately detect kidney stones from endoscopic video data. The model’s ability to classify the predominant stone composition could further be enhanced by providing high-quality video data for training.
22.	Noble PA et al., 2024, USA [52].	To develop a successful predictive model for stone removal and treatment complications in patients undergoing ESWL and laser ureterorenoscopy (URS).	The researchers utilized ANN models trained on a dataset comprising 15,126 ESWL and 2116 URS patient records. For URS, the average prediction accuracy was 89.0%, and for SWL stone removal and treatment complications, it was 95.0% and 84.8%, respectively. SWL and URS have AUC scores of 74.7% and 62.9%, respectively, and 77.2% and 78.9%, respectively.	The created models were integrated into a Stone Decision Engine web tool to help healthcare providers choose the best interventions based on patient data, and they demonstrated moderate to high accuracy in predicting outcomes for both therapy types.
23.	Chmiel JA et al., 2024, Canada [53].	To explore the application of machine learning techniques to predict the composition of urinary stones, given that stone composition is related to physiological parameters during its formation.	The primary objective was to develop predictive models that could accurately classify the composition of urinary stones, which commonly include calcium oxalate, uric acid, and calcium phosphate. The kappa score was utilized to evaluate model performance, and the impact of each predictor variable was analyzed.	The findings suggest that using machine learning for clinical data interpretation can predict urinary stones composition. By understanding the composition of urinary stones, clinicians can recommend targeted dietary modifications and pharmacological therapies that are more likely to be effective for individual patients.
24.	Cumpanas AD et al., 2024, California [54].	An innovative approach utilizing an automated AI algorithm designed for CT scans in order to streamline the process of stone volume determination.	The scalene, pro-late, and oblate ellipsoid formulas’ estimated volumes were then compared with the AI-calculated index stone volume and the ground truth volume. The authors emphasize that the conventional methods, which typically rely on linear measurements or the ellipsoid formula, can lead to significant inaccuracies.	The authors report that their AI algorithm significantly outperforms traditional methods, demonstrating a marked reduction in interobserver variability, providing accurate, precise, and time-efficient stone volume measurements.
**CHATBOT COMPARISON**				
25.	Panthier F et al., 2024, France [55].	The study aimed to investigate the capacity of four large language models to write a systematic review on the subject of pulsed-Thulium:YAG laser for lithotripsy.	The four AI-generated reviews were compared to a human-written review. A list of ten “checkpoints” was defined by the first author and independently reviewed by the senior author. These checkpoints related to aspects of the laser’s technology and specific details of the results for lithotripsy. The blinded manuscripts were then submitted to nine participants with varying levels of expertise in urology, who evaluated the presence/absence of the checkpoints and made three subjective assessments: the overall quality and clarity of the manuscripts and an overall ranking from 1 to 5.	The human-written systematic review was objectively and subjectively more accurate than the AI-generated ones, with higher accuracy in highly technical topics. Among the evaluated reviews, the one produced by ChatGPT-4 had the highest scores regarding subjective and objective accuracy.
26.	Şahin MF et al., 2024, Turkey [56].	The purpose of this study was to evaluate and compare the quality and comprehensibility of the responses that were generated by five different AI chatbots: ChatGPT-4, Claude, Mistral, Google PaLM, and Grok, in response to the questions that were searched most frequently regarding kidney stones.	A distinct set of 25 frequently searched terms was given to each AI chatbot as input. DISCERN, the Flesch-Kincaid Grade Level, the Flesch-Kincaid Reading Ease, and the Patient Education Materials Assessment Tool for Printable Materials were used to evaluate the replies.	Of the five chatbots, Grok was the easiest to read and understand, whereas GPT-4 had the most complicated linguistic structure. Claude’s kidney stones text quality was the best. Chatbot technology has the potential to enhance and simplify healthcare content.
27.	Musheyev D et al., 2024, USA [57].	To assess the accuracy and reliability of health information about kidney stones disseminated by various AI chatbots.	Four AI chatbots (ChatGPT version 3.5, Perplexity, Chat Sonic, and Bing AI) were trained using the most popular kidney stone Internet queries from Google Trends and headers from the National Institute of Diabetes and Digestive and Kidney Diseases website. The quality (DISCERN instrument, which ranges from 1 low to 5 high), understandability, and actionability (PEMAT, which ranges from 0% to 100%) of the chatbot outputs were evaluated using validated tools.	In general, AI chatbots did not propagate false information and offered high-quality consumer health information. However, the given information was beyond the recommended reading level for consumer health information.
28.	Touma NJ et al., 2024, Canada [58].	To evaluate Chat GPT-4’s performance on a multiple-choice test that simulates the Canadian Urology Board Exam.	On the MCQ exam, Chat GPT-4 had a score of 46%, with particularly low scores in oncology (35%) and trauma/reconstruction (17%), while the mean and median scores of urology residents who were graduating were 62.6% and 62.7%, respectively.	Chat GPT-4 had a low performance in the simulated exam. As these models develop and are trained on more urology content, ongoing evaluations of generative AI’s potential are required.
29.	Altıntaş E et al., 2024, Turkey [59].	To assess how effectively AI chatbots adhere to the EAU guidelines when providing recommendations for urolithiasis management.	Perplexity and Chat GPT-4.0’s average scores were 4.68 and 4.80, respectively, and both were considerably different from Bing and Bard’s results.	Chat GPT-4.0 and Perplexity were found to adhere well to EAU guideline recommendations. Updates to AI systems to maintain their accuracy and reliability are required to use them in improving urolithiasis patient outcomes.

## Data Availability

All data are provided in this manuscript.
